# Mortality Rates in Trials of Subjects With Type 2 Diabetes

**DOI:** 10.1161/JAHA.111.000059

**Published:** 2012-02-20

**Authors:** Ebrahim Barkoudah, Hicham Skali, Hajime Uno, Scott D. Solomon, Marc A. Pfeffer

**Affiliations:** Cardiovascular Division, Dana Farber Cancer Institute, Boston, MA (E.B., H.S., S.D.S., M.A.P.); General Medicine Division, Brigham and Women's Hospital, Dana Farber Cancer Institute, Boston, MA (E.B.); Department of Biostatistics and Computational Biology, Dana Farber Cancer Institute, Boston, MA (H.U.)

**Keywords:** type 2 diabetes, chronic kidney disease, mortality, controlled clinical trials, randomized

## Abstract

**Background:**

In randomized controlled trials (RCTs) of subjects with type 2 diabetes mellitus, mortality rates vary substantially. We sought to examine the inclusion and exclusion criteria of these RCTs to explore relationships with mortality.

**Methods and Results:**

MEDLINE database was searched from August 1980 through March 2011. Selection criterion included published RCTs of adults with type 2 diabetes mellitus of at least 1000 patients, reporting all-cause mortality and having follow-up duration of at least 1 year. Twenty-two trials were eligible. Annualized mortality rates were derived. Inclusion and exclusion criteria were tabulated for each trial. Trials were categorized in 4 groups according to annual mortality rates: <1, ≥1 to <2, ≥2 to <4, and ≥4 per 100 patient-years. The analysis cohort included 91842 patients and 6837 deaths. Mortality rates ranged from 0.28 to 8.24 per 100 patient-years. Patients enrolled in the highest mortality category were more likely to be older and had longer diabetes duration and higher blood pressure. The selection for hypertension was common in the low- as well as high-mortality trials. Although the mortality rates were higher in RCTs with prior cardiovascular morbidity, the selection for chronic kidney disease—defined by either higher serum creatinine or lower estimated glomerular filtration rate and/or the presence of proteinuria—was associated with the highest mortality rates.

**Conclusions:**

In this analysis of RCTs of type 2 diabetes mellitus, a 29-fold difference in annualized mortality was observed. In these RCTs, selection for renal disease, defined by either decline in renal function or presence of proteinuria, portends important mortality risk. **(*J Am Heart Assoc*. 2012;1:8-15.)**

**Clinical Trial Registration:**

URL: http://www.ClinicalTrials.gov. Unique identifier: NCT00303979

## Background

Type 2 diabetes mellitus (T2DM) increases the risk of premature morbidity and mortality in the community and cardiovascular disease (CVD) is the leading cause of death in individuals with diabetes.^1,2^ The burden of diabetes-related CVD is likely to continue to expand with the increasing incidence of diabetes in the population.^3^

It is clear from epidemiologic studies that concomitant diabetes augments the hazard associated with other risk factors for developing CVD, including hypertension, hyperlipidemia, and renal impairment.^4,5^ Similarly, in patients with established CVD, diabetes portends a greater risk of worse outcomes especially when associated complications such as nephropathy, retinopathy, and, possibly, neuropathy are present. Conversely, in patients whose only risk factor is diabetes, the population attributable risk of death may not be very high.^6^

In contrast to epidemiologic studies, which provide the most accurate assessment of disease burden in population, randomized controlled trials (RCTs) use inclusion and exclusion criteria to target a specific subpopulation and provide an insight to contributions of comorbidities on mortality in a selected population. Although the specific selection criteria distort the disease proportion in the population, including patients who fulfill specific selection criteria can augment the overall morbidity and mortality risk. Across trials, the selection or exclusion of a specific risk profile offers a diverse sample that extends from low mortality to those with the extreme hazard.

In RCTs of T2DM patients, while any cardiovascular or renal diseases increase mortality in diabetes, patients without evidence of such would be expected to experience fewer complications.^7^ Furthermore, annualized death rates can characterize the risk in diabetes populations across trials. We used data from recent RCTs in T2DM to further examine the relationship between cardiovascular risk factors and renal disease and its complications and how this interplay augments the risk of death.

## Methods

### Study Selection and Search Strategy

The selection criteria included RCTs of adults with T2DM of at least 1000 patients, reporting all-cause mortality results, and with a minimum follow-up duration of 1 year. We excluded trials that selected patients with acute coronary syndrome or end-stage renal disease.

**Figure 1.**
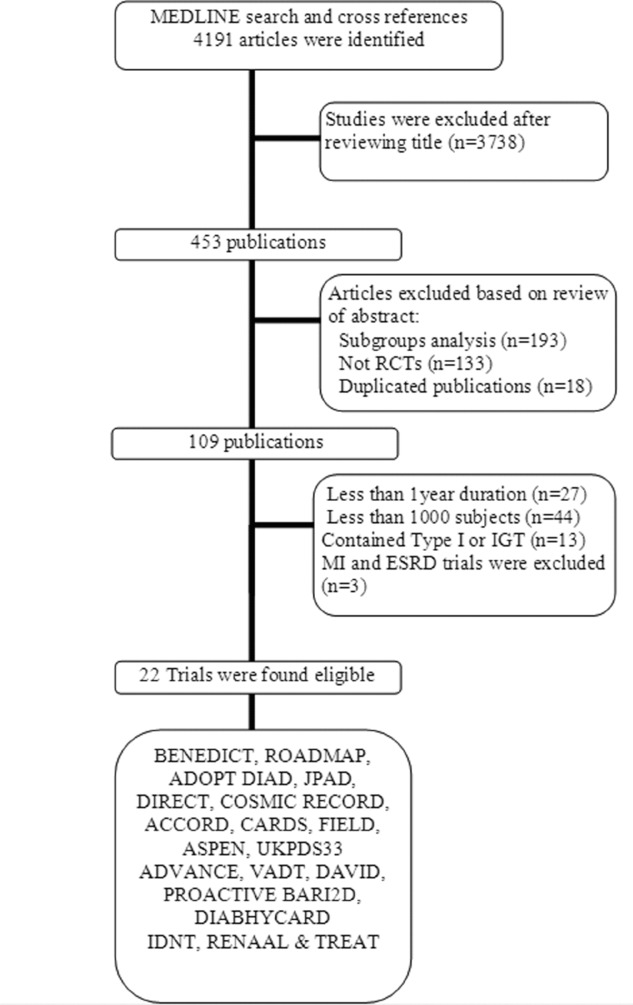
Literature flow.

The search included the following terms: MeSH major topic: diabetes mellitus type 2 treatment; Limits: Humans, RCT, English, and 19+ years adult. The MEDLINE database was searched for studies using these eligibility criteria between August 1980 and March 2011. All references were examined to attempt identify additional trials. The initial search generated 4191 publications. After reviewing titles and abstracts (Figure 1), and applying prespecified criteria, 22 studies ^8–29^ were eligible (Table 1).

**Table 1.** Eligible Studies Sorted by Publication Date

Acronyms	Trial	Publication
UKPDS33	UK Prospective Diabetes Study ^8^	*Lancet*. 1998;352:837–853.

IDNT	Irbesartan in Patients with Nephropathy due to Type 2 Diabetes ^9^	*N Engl J Med*. 2001;345:851–860.

RENAAL	Reduction of Endpoints in NIDDM with the Angiotensin II Antagonist Losartan ^10^	*N Engl J Med*. 2001;345:861-869.

DIABHYCAR	Non–insulin-dependent DIABetes, Hypertension, microalbuminuria or proteinuria, CARdiovascular events, and Ramipril ^11^	*BMJ*. 2004;328:495–500.

DAVID	Atherosclerotic Vascular Disease in Diabetics ^12^	*Eur Heart J*. 2004; 25:1845–1852.

CARDS	Collaborative Atorvastatin Diabetes Study ^13^	*Lancet*. 2004;364:685–696.

BENEDICT	Bergamo Nephrologic Diabetes Complications Trial ^14^	*N Engl J Med*. 2004;351:1941-1951.

COSMIC	Comparative Outcomes Study of Metformin Intervention versus Conventional ^15^	*Diabetes Care*. 2005;28:539–543.

PROactive	PROspective pioglitAzone Clinical Trial In macroVascular Events ^16^	*Lancet*. 2005;366:1279–1289.

FIELD	Fenofibrate Intervention and Event Lowering in Diabetes ^17^	*Lancet*. 2005;366:1849–1861.

ASPEN	The Atorvastatin Study for Prevention of Coronary Heart Disease Endpoints in Non-Insulin-Dependent Diabetes Mellitus ^18^	*Diabetes Care*. 2006;29:1478–1485.

ADOPT	A Diabetes Outcome Progression Trial ^19^	*N Engl J Med*. 2006;355:2427–2443.

ACCORD	Action to Control Cardiovascular Risk in Diabetes ^20^	*N Engl J Med*. 2008;358:2545–2559.

ADVANCE	Action in Diabetes and Vascular Disease ^21^	*N Engl J Med*. 2008;358:2560-2572.

DIRECT-2	DIabetic REtinopathy Candesartan Trial ^22^	*Lancet*. 2008;372:1385–1393.

JPAD	Japanese Primary Prevention of Atherosclerosis With Aspirin for Diabetes ^23^	*JAMA*. 2008;300:2134–2141.

VADT	Veterans Affairs Diabetes Trial ^24^	*N Engl J Med*. 2009;360:129–139.

DIAD	Detection of Ischemia in Asymptomatic Diabetics ^25^	*JAMA*. 2009;301:1547–1555.

RECORD	Rosiglitazone Evaluated for Cardiac Outcomes and Regulation of Glycemia in Diabetes ^26^	*Lancet*. 2009;373:2125–2135.

BARI2D	Bypass Angioplasty Revascularization Investigation 2 Diabetes ^27^	*N Engl J Med*. 2009;360:2503–2515.

TREAT	Trial to Reduce Cardiovascular Events With Aranesp Therapy ^28^	*N Engl J Med*. 2009;361:2019–2032.

ROADMAP	The Randomized Olmesartan and Diabetes Microalbuminuria Prevention ^29^	*N Engl J Med*. 2011;364:907–917.

### Data Extraction, Synthesis, and Analysis

In RCTs that met our criteria, we calculated the all-cause mortality rate for the overall population, regardless of treatment allocation. Patient-years were estimated in each trial by multiplying the sample size by the average follow-up time (median was used when mean was not reported). Annualized mortality incidence rates were derived accordingly by dividing the total number of deaths by total patient-years, from all treatment arms, and expressed per 100 patient-years. Because most of these trials were not designed or powered to study the effect on all-cause mortality, we used the overall mortality rate regardless from intervention allocation.

Inclusion and exclusion criteria were obtained for each trial. When available, the selection—or exclusion—for hypertension, CVD, elevated serum creatinine or estimated glomerular filtration rate<60 mL/min/1.73 m^2^, or the presence of proteinuria were tabulated. When a specific inclusion/exclusion criterion was not specified, we considered it a permitted condition. RCTs were sorted by ascending mortality rates and then categorized in 4 groups: <1, ≥1 to <2, ≥2 to <4, and ≥4 deaths per 100 patient-years. Within each mortality group, annualized mortality rates were derived by dividing the sum of deaths by total patient-years of group's trials and express the product by 100 patient-years.

Within each category, every trial was attributed a weight according to its sample size. Weighted values (ie, weighted means and corresponding 95% confidence intervals) for baseline characteristics were calculated accordingly.^30^ When available, we obtained the mean or median of the descriptive characteristics of the population: age, sex, body mass index, duration of diabetes, total cholesterol, low-density lipoprotein cholesterol, systolic blood pressure, serum creatinine, smoking status along with the rates of retinopathy, hypertension, history of CVD, and the presence of proteinuria defined by any abnormal excretion of albumin or protein.

## Results

**Figure 2.**
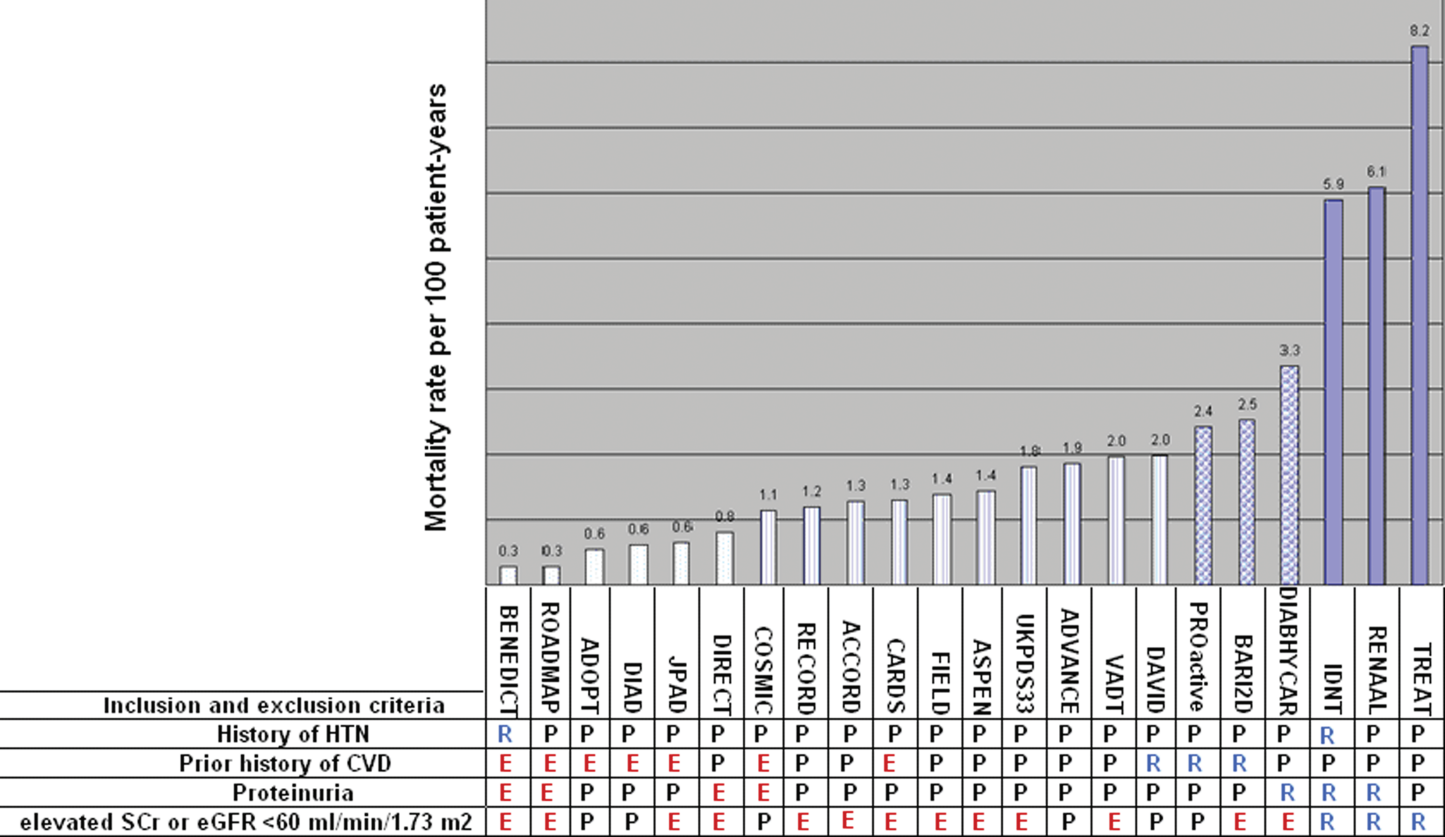
All eligible trials according to ascending death rate. R: condition required; P: condition permitted; E: condition excluded; E: excluded as directly stated or no mention of baseline MI, HF, or Stroke for prior history of CVD. Also excluded for serum creatinine >1.5 or >1.7 per trial's protocol.

Our analysis included 91742 patients and 6837 deaths (7.5%). The overall mortality rate in the entire population was 1.83 deaths per 100 patient-years and varied widely from 0.28 to 8.24 deaths per 100 patient-years across the selected trials (Figure 2 and Table 2).

**Table 2.** Summary of Baseline Characteristics of All Eligible Trials Arranged by Ascending Death Rate

	BENEDICT	ROADMAP	ADOPT	DIAD	JPAD	DIRECT	COSMIC	RECORD	ACCORD	CARDS	FIELD
Patients	1204	4447	4360	1123	2539	1905	8732	4447	10251	2838	9795

Follow-up (y)	3.6 *	3.2 *	4.0*	4.8	4.4*	4.7	1.0	5.5	3.5	3.9*	5.0*

Death *n* (%)	12 (1)	41 (1)	96 (2)	33 (3)	72 (3)	72 (4)	100 (1)	293 (7)	460 (4)	143 (5)	679 (7)

Follow-up in patient-years	4334	14230	17440	5390	11095	8954	8732	24459	35879	11068	48975

Death rate†	0.28	0.29	0.55	0.61	0.65	0.80	1.15	1.20	1.28	1.29	1.39

Age	62.0	57.7	56.8	60.8	64.5	56.8	58.4	58.4	62.2	61.6	62.2

Diabetes duration	7.9	6.1		8.6	7.0*	8.8	4.9	7.1	10.0*	7.8	5.0*

Female, sex, (%)	44	54	42	47	45	50	51	48	39	32	37

BMI (kg/m^2^)	31.2	31.0	32.1	31.1	24.0	29.4		31.5	32.2	29.0	29.8*

CVD (%)				9	0			31	35	0	22

Hypertension (%)	100		78		58	62		80	85	84	57

Smoker (%)	12	19	15	10	21	15		16	14	23	9

SBP (mmHg)	151	141	133	132	135	123		139	136	144	141

Total cholesterol (mg/dL)	210	207	204*		201	205			183	209	193

LDL (mg/dL)	162	125	120*	114				128	105	117	119

HbA_1c_ (%)	5.8	7.6	7.7	7.1	7.0	8.2		7.9	8.3	7.8	6.9*

SCr (mg/dL)	0.9	0.9		1.0	0.8	1.0		0.7	0.9	1.2	0.9

Retinopathy (%)				15	15	100		10	34	30	8

Proteinuria (%)	0	0	16	24	18	0		19	30	17	22

	ASPEN	UKPDS33	ADVANCE	VADT	DAVID	PROACTIVE	BARI2D	DIABHYCAR	IDNT	RENAAL	TREAT
Patients	2410	3867	11140	1791	1209	5238	2368	4912	1715	1513	4038

Follow-up (y)	4.0*	10.0*	5.0*	5.6*	2.0	2.9	5.3	4.0*	2.6	3.4	2.4

Death *n* (%)	138 (6)	702 (18)	1031 (9)	197 (11)	48 (4)	363 (7)	316 (13)	658 (13)	263 (15)	313 (21)	807 (20)

Follow-up in patient-years	9640	38670	55700	10030	2418	15033	12550	19648	4459	5144	9792

Death rate†	1.43	1.82	1.85	1.96	1.99	2.41	2.52	3.35	5.90	6.08	8.24

Age	61.1	53.3	66.0	60.4	64.2	61.7	62.4	65.0	59.0	60.0	68.0

Diabetes duration	8.0	0.3	7.9	11.5		8.0*	10.4	9.8	14.9*	15.0*	15.4*

Female, sex, (%)	34	39	43	3	27	34	30	30	34	37	57

BMI (kg/m^2^)	28.9	27.5	28.0	31.2	28.4	30.8	31.7	29.2	30.8	29.4	30.2*

CVD (%)		2	32	40	100	100	100	24	29	47	65

Hypertension (%)	55			72	38	76	83	56	100	96	

Smoker (%)	12	31	14	16	29	14	13	15		18	5

SBP (mmHg)	133	135	145	132	147	143	132	145	159	153	136*

Total C (mg/dL)	193	209	201	174			169		229	228	163*

LDL (mg/dL)	114*	135	120	101		112*	96			137	85*

HbA_1c_ (%)	7.6	7.1	7.5	9.4		7.9*	7.7	7.8	8.2	8.4	6.9*

SCr (mg/dL)		0.9*	1.0	1.0		0.9*	1.0	1.0	1.7	1.9	1.9*

Retinopathy (%)		36	7				3	5	66	64	47

Proteinuria (%)¶		9	31	48		44	33	100	100	100	100

BMI indicates body mass index is the weight in kilograms divided by the square of the height in meters; CVD, cardiovascular disease composite; HbA1c, hemoglobin A1c; LDL, low-density lipoprotein; SBP, systolic blood pressure; SCr, serum creatinine; smoker: donates current smoker status.

* Value was reported as median, otherwise mean;

† Per 100 patient-years;

¶ Proteinuria when available.

To convert the values for cholesterol to millimoles per liter, multiply by 0.02586. To convert the values for creatinine to milligrams per deciliter, divide by 88.4.

Values were approximated to the hundredths place for follow-up time, death rate, and to the tenth place for follow-up time, age, diabetes duration, female gender, BMI, A1c. SCr. CVD, hypertension, smoker, SBP, Total C, LDL, retinopathy and micro/macroalbuminuria values were rounded off to the nearest one place.

Total C: total cholesterol.

All RCTs included additional risk factors other than diabetes in their inclusion criteria. Hypertension was a common inclusion criterion for low- and high-mortality trials. Mortality rates were the lowest in primary prevention trials and those for which hypertension was the only additional inclusion criterion (BENIDICT, DIRECT, JPAD, and ROADMAP). These trials, excluding JPAD, shared an exclusion criterion of proteinuria at baseline. Studies selecting for the presence of CVD other than hypertension (DAVID, PROACTIVE, and BARI2D) had higher mortality than those permitting CVD in their inclusion criteria (RECORD, ACCORD, FIELD, UKPDS, ADVANCE, and VADT). Moreover, trials in which inclusion required elevated serum creatinine values (mostly >1.5 mg/dL) or estimated glomerular filtration rate<60, or/and evident proteinuria (DIABHYCAR, IDNT, RENAAL, and TREAT) showed the highest mortality rates.

Mortality, across trials, was associated with certain characteristics: serum creatinine, proteinuria prevalence, age, diabetes duration, and systolic blood pressure averages (Table 3). Furthermore, subjects in the lower mortality category were younger (59 years), had lower serum creatinine averages (0.9 mg/dL), and proteinuria prevalence (9%). Conversely, subjects enrolled in the highest mortality trials where more likely to be older (64.2 years); have longer diabetes duration (15.2 years); and have higher systolic blood pressure (145 mmHg), serum creatinine averages (1.8 mg/dL), and proteinuria prevalence (100%).

**Table 3.** Comparison of Baseline Characteristics in 4 Categories

Category	<1	≥1 to <2	≥2 to <4	≥4 to <10	All
Mortality rate¶	0.53	1.54	2.83	7.13	1.83

No. of trials	6	10	3	3	22

No. of patients	15578	56480	12518	7266	91842

Total patient-years	61444	245570	47231	19395	373641

Death (*n*)	326	3791	1337	1383	6837

Follow-up (y)* [no. of reporting trials]	4.3 [6]	5.4 [10]	4.0 [3]	2.7 [3]	4.0 [22]

Female % [no. of reporting trials]	47 [6]	40 [10]	32 [3]	47 [3]	40 [22]
(95% confidence interval)	(47–48)	(39–40)	(31–32)	(46–49)	

Age *[no. of reporting trials]	59.0 [6]	61.4 [10]	63.1 [3]	64.2 [3]	61.6 [22]
(95% confidence interval)	(58.7–59.3)	(61.2–61.6)	(62.9–63.4)	(63.9–64.6)	

DM t* (y) [no. of reporting trials]	7.2 [5]	6.8 [9]	9.2 [3]	15.2 [3]	8.9 [20]
(95% confidence interval)	(7.0–7.4)	(6.7–6.9)	(8.9–9.4)	(14.9–15.5)	

HbA_1c_ * [no. of reporting trials]	7.3 [6]	7.6 [8]	7.8 [3]	7.5 [3]	7.4 [20]
(95% confidence interval)	(7.3–7.4)	(7.6–7.7)	(7.8–7.9)	(7.5–7.6)	

BMI * [no. of reporting trials]	30.0 [6]	29.8 [9]	30.3 [3]	30.2 [3]	29.9 [21]
(95% confidence interval)	(29.8–30.2)	(29.7–29.9)	(30.2–30.5)	(29.9–30.4)	

HTN %* [no. of reporting trials]	73 [4]	72 [7]	69 [3]	97 [2]	73 [16]
(95% confidence interval)	(72–74)	(71–72)	(68–70)	(96–98)	

CVD % * [no. of reporting trials]	3 [2]	28 [8]	70 [3]	53 [3]	31 [16]
(95% confidence interval)	(2–3)	(28–29)	(70–71)	(52–54)	

Smokers% *[no. of reporting trials]	16 [6]	15 [9]	14 [3]	9 [2]	16 [20]
(95% confidence interval)	(16–17)	(15–16)	(13–15)	(8–9)	

SBP *(mmHg) no. of reporting trials]	136 [6]	140 [9]	142 [3]	145 [3]	139 [21]
(95% confidence interval)	(135–136)	(139–140)	(140–142)	(144–146)	

SCr * (mg/dL) [no. of reporting trials]	0.9 [5]	0.9 [7]	1.0 [2]	1.8 [3]	1.0 [17]
(95% confidence interval)	(0.9–0.9)	(0.9–0.9)	(0.9–1.0)	(1.8–1.9)	

Total C*(mg/dL) [no. of reporting trials]	205 [5]	195 [7]	169 [1]	192 [2]	195 [15]
(95% confidence interval)	(203–206)	(193–196)	(168–171)	(191–194)	

LDL*(mg/dL) [no. of reporting trials]	126 [4]	117 [8]	107 [2]	99 [2]	116 [16]
(95% confidence interval)	(126–127)	(116–118)	(106–108)	(98–101)	

Proteinuria %*[no. of reporting trials]	9 [6]	21 [7]	52 [3]	100 [3]	32 [19]
(95% confidence interval)	(8–9)	(20–22)	(51–53)	(100–100)	

Retinopathy % *[no. of reporting trials]	43 [3]	18 [6]	3 [2]	55 [3]	23 [14]
(95% confidence interval)	(42–45)	(18–19)	(2–3)	(54–56)	

BMI indicates body mass index is the weight in kilograms divided by the square of the height in meters; CVD, sum of any cardiovascular disease incidence; DM t: diabetes mellitus duration, time; HbA_1c_, hemoglobin A_1c_, Total C: total cholesterol; LDL, low-density lipoprotein; SBP, systolic blood pressure; SCr, serum creatinine; Smoker, donates current smoker status.

* Utilizing weight of patient sample; [] No. of contributed trials in each category;

¶ Per 100 patient-years; percentage of subjects with micro/macroalbuminuria or proteinuria in trial as defined by publication.

To convert the values for cholesterol to millimoles per liter, multiply by 0.02586. To convert the values for creatinine to milligrams per deciliter, divide by 88.4.

The body mass index is the weight in kilograms divided by the square of the height in meters.

Values were approximated to the hundredths place for death rate and to the tenth place for follow-up time, age, diabetes duration, BMI, HbA_1c_. SCr. CVD, hypertension, smoker, SBP, Total C, LDL, retinopathy, and microalbuminuria incidences were rounded off to the tens place.

Lower low-density lipoprotein averages and current smoking status were observed with higher mortality rates (126, 117, 107, 99 mg/dL and 16%, 15%, 14%, and 9%, respectively, along the categories). In addition, we observed variation in body mass index and glycated hemoglobin averages across mortality and were highest in the trials with CVD trials. Moreover, hypertension prevalence was highest in the highest mortality trials category (97%) and ranged 69% to 73% across all other mortality categories. Similarly, reporting retinopathy varied in consistency across mortality groups with higher presence in the highest mortality category. Gender type was not associated with mortality in our analysis.

## Discussion

Patients with diabetes mellitus enrolled in RCTs do not represent a homogenous population.^31^ Different inclusion criteria, according to study aim, influence baseline characteristics of the enrolled subjects and subsequently the risk profile and observed outcomes. As a result, the heterogeneity of this population is translated into a wide range of death rates. The term “diabetes risk” in and of itself does not differentiate those with the highest death risk in the spectrum.

Our report highlights the risk of death in subjects with diabetes, with an approximately 30-fold difference in annualized mortality rate across the range of trials we examined. Hypertension was not associated with higher mortality rate and selection for hypertension specifically did not discriminate those at higher risk of death. Furthermore, in RCTs of subjects with diabetes, the selection for increased urinary protein excretion or/and elevated serum creatinine was particularly and closely associated with increased risk of death.

Our study is consistent with prior reports describing the risk associated with nephropathy in diabetes. UKPDS 64 data described mortality rates similar to our data with a stepwise increase in death rate on the progression from normoalbunimura to albuminuria to decline in kidney function.^32^ Although multiple studies demonstrated that baseline characteristics affect mortality in diabetic subjects^33,34^, our descriptive report shows that chronic kidney disease (CKD) in diabetes is associated with major mortality risk.

In addition to diabetes and CKD, TREAT also required hemoglobin level ≤11.0 g/dL as an inclusion criterion. This additional eligibility requirement would be expected to be associated with a greater risk of death.^35^ Additionally, other detectable serum cardiac biomarkers were shown to be associated with even higher risk of death ^36^ and could potentially be used for further risk stratification and/or selection of specific populations for clinical trials.^37^

The Emerging Risk Factors Collaboration showed that diabetes increases the risk of vascular and nonvascular mortality causes.^38^ The report also demonstrated that the risk death increased with estimated glomerular filtration rate decline. Our study illustrates that any degree of proteinuria in diabetes is associated with a higher death rate. Risk stratification by evidence of kidney disease, determined by renal function or proteinuria, should be emphasized in diabetic subjects given its major impact on morbidity and mortality.^39^

Most clinical trials, by design, augment the studied risk in clinical trials and eventually increase endpoint rates. In our cohort of all subjects with type 2 diabetes, each trial contains an attributed risk in its inclusion. Although hypertension is an element of the cardiovascular continuum risk profile in diabetes, its mortality augmentation was not evident in our cohort especially in the absence of proteinuria and did not provide further risk discrimination. Recent analysis concluded the similar finding.^40^ Although the diagnosis of hypertension was common across all morality categories, the actual weighted systolic blood pressure average was greater in the highest mortality trials.

Within our cohort of subjects with T2DM participants in RCTs, we did not observe an increase of other “traditional” risk factors along with increasing mortality.^41^ Moreover, variation of certain characteristic risk factors was seen across the selected trials; for example, body mass index and glycated hemoglobin weighted average values showed a disparity across the arbitrary categories and likely due to the selection processes in these trials. Notably, current smoking and low-density lipoprotein weighted averages were lower in higher mortality categories. Although the lower prevalence of smoking is probably related to the inclusion criteria, reports from cohorts of CKD and heart failure demonstrated that lower cholesterol correlates with higher mortality what is described as “reverse epidemiology” in other population of patients with advanced disease.^42^ Moreover, the presence of CKD matched higher presence of hypertension and retinopathy. This matching increase is probably explained by the bidirectional influence between the vasculature and the kidney along with the end-organ damage of both systems simultaneously.^43^

Our report was limited by the lack of individual patient data to allow detailed analyses of baseline characteristics and risk of death. Baseline characteristics were obtained from the publication as averages; not all baseline characteristics were reported in every trial; we also used overall prevalence of CVDs in our analysis and not its subtypes. Furthermore, we did not account for the medications and therapeutic interventions during trials. We examined overall mortality in the analyzed trials and did not take into account randomized treatment arms and treatment effects on mortality, albeit these were generally neutral. Although we also did not account for potential secular decline in mortality rates over time, 21 of our 22 trials in our analysis were published in the past 10 years. The strength of this report includes the review of numerous baseline demographics and examination of a large cohort. Moreover, the extensive literature search that was performed makes publication selection bias less likely. Our report focuses on comparing the exclusion and inclusion criteria and demonstrating the contrast in the clinical trials population as selective cohort to supplement for epidemiologic studies, which have critical estimates of demographics. Furthermore, the truncated distribution of the population demographics was dictated by the trials selection criteria and probably resulted in a less pronounced relationship between demographics and outcomes.

Mortality has a broad range of representation in trials of type 2 diabetes subjects. Moreover, diabetes trials with nephropathy selection had the highest death rates. We conclude that the selection for CKD, defined by either decline in renal function or presence of proteinuria, augmented the death risk in diabetes.

## References

[1] Roglic G, Unwin N, Bennett PH, Mathers C, Tuomilehto J, Nag S, Connolly V, King H (2005). The burden of mortality attributable to diabetes: realistic estimates for the year 2000. Diabetes Care.

[2] Sowers JR, Lester MA (1999). Diabetes and cardiovascular disease. Diabetes Care.

[3] Huang ES, Basu A, O'Grady M, Capretta JC (2009). Projecting the future diabetes population size and related costs for the U.S. Diabetes Care.

[4] Panzram G (1987). Mortality and survival in type 2 (non-insulin-dependent) diabetes mellitus. Diabetologia.

[5] Buse JB, Ginsberg HN, Bakris GL, Clark NG, Costa F, Eckel R, Fonseca V, Gerstein HC, Grundy S, Nesto RW, Pignone MP, Plutzky J, Porte D, Redberg R, Stitzel KF, Stone NJ (2007). Primary prevention of cardiovascular diseases in people with diabetes mellitus: a scientific statement from the American Heart Association and the American Diabetes Association. Circulation.

[6] Boyko EJ, Meigs JB (2011). Does diabetes always confer coronary heart disease risk equivalent to a prior myocardial infarction?: implications for prevention. Diabetes Care.

[7] Ingelfinger JR (2011). Preemptive olmesartan for the delay or prevention of microalbuminuria in diabetes. N Engl J Med.

[8] UK Prospective Diabetes Study (UKPDS) Group (1998). Intensive blood-glucose control with sulphonylureas or insulin compared with conventional treatment and risk of complications in patients with type 2 diabetes (UKPDS 33). Lancet.

[9] Lewis EJ, Hunsicker LG, Clarke WR, Berl T, Pohl MA, Lewis JB, Ritz E, Atkins RC, Rohde R, Raz I, Collaborative Study Group (2001). Renoprotective effect of the angiotensin-receptor antagonist irbesartan in patients with nephropathy due to type 2 diabetes. N Engl J Med.

[10] Brenner BM, Cooper ME, de Zeeuw D, Keane WF, Mitch WE, Parving HH, Remuzzi G, Snapinn SM, Zhang Z, Shahinfar S (2001). Effects of Losartan on renal and cardiovascular outcomes in patients with type 2 diabeyes and nephropathy. N Engl J Med.

[11] Marre M, Lievre M, Chatellier G, Mann JF, Passa P, Ménard J (2004). Effects of low dose ramipril on cardiovascular and renal outcomes in patients with type 2 diabetes and raised excretion of urinary albumin: randomised, double blind, placebo controlled trial (the DIABHYCAR study). BMJ.

[12] Neri Serneri GG, Coccheri S, Marubini E, Violi F (2004). Picotamide, a combined inhibitor of thromboxane A2 synthase and receptor, reduces 2-year mortality in diabetics with peripheral arterial disease: the DAVID study. Eur Heart J.

[13] Colhoun HM, Betteridge DJ, Durrington PN, Hitman GA, Neil HA, Livingstone SJ, Thomason MJ, Mackness MI, Charlton-Menys V, Fuller JH (2004). Primary prevention of cardiovascular disease with atorvastatin in type 2 diabetes in the Collaborative Atorvastatin Diabetes Study (CARDS). Lancet.

[14] Ruggenenti P, Fassi A, Ilieva AP, Bruno S, Iliev IP, Brusegan V, Rubis N, Gherardi G, Arnoldi F, Ganeva M, Ene-Iordache B, Gaspari F, Perna A, Bossi A, Trevisan R, Dodesini AR, Remuzzi G (2004). Preventing microalbuminuria in type 2 diabetes. N Engl J Med.

[15] Cryer DR, Nicholas SP, Henry DH, Mills DJ, Stadel BV (2005). Comparative outcomes study of metformin intervention versus conventional approach. Diabetes Care.

[16] Dormandy JA, Charbonnel B, Eckland DJ, Erdmann E, Massi-Benedetti M, Moules IK, Skene AM, Tan MH, Lefèbvre PJ, Murray GD, Standl E, Wilcox RG, Wilhelmsen L, Betteridge J, Birkeland K, Golay A, Heine RJ, Korányi L, Laakso M, Mokán M, Norkus A, Pirags V, Podar T, Scheen A, Scherbaum W, Schernthaner G, Schmitz O, Skrha J, Smith U, Taton J (2005). Secondary prevention of macrovascular events in patients with type 2 diabetes in the PROactive Study (PROspective pioglitAzone Clinical Trial In macroVascular Events). Lancet.

[17] Keech A, Simes RJ, Barter P, Best J, Scott R, Taskinen MR, Forder P, Pillai A, Davis T, Glasziou P, Drury P, Kesäniemi YA, Sullivan D, Hunt D, Colman P, d'Emden M, Whiting M, Ehnholm C, Laakso M (2005). Effects of long-term fenofibrate therapy on cardiovascular events in 9795 people with type 2 diabetes mellitus (the FIELD study): randomised controlled trial. Lancet.

[18] Knopp RH, d'Emden M, Smilde JG, Pocock SJ (2006). Efficacy and safety of atorvastatin in the prevention of cardiovascular end points in subjects with type 2 diabetes: the Atorvastatin Study for Prevention of Coronary Heart Disease Endpoints in non-insulin-dependent diabetes mellitus (ASPEN). Diabetes Care.

[19] Kahn SE, Haffner SM, Heise MA, Herman WH, Holman RR, Jones NP, Kravitz BG, Lachin JM, O'Neill MC, Zinman B, Viberti G, ADOPT Study Group (2006). Glycemic durability of rosiglitazone, metformin, or glyburide monotherapy. N Engl J Med.

[20] Gerstein HC, Miller ME, Byington RP, Goff DC, Bigger JT, Buse JB, Cushman WC, Genuth S, Ismail-Beigi F, Grimm RH, Probstfield JL, Simons-Morton DG, Friedewald WT (2008). Effects of intensive glucose lowering in type 2 diabetes. N Engl J Med.

[21] Patel A, MacMahon S, Chalmers J, Neal B, Billot L, Woodward M, Marre M, Cooper M, Glasziou P, Grobbee D, Hamet P, Harrap S, Heller S, Liu L, Mancia G, Mogensen CE, Pan C, Poulter N, Rodgers A, Williams B, Bompoint S, de Galan BE, Joshi R, Travert F (2008). Intensive blood glucose control and vascular outcomes in patients with type 2 diabetes. N Engl J Med.

[22] Sjølie AK, Klein R, Porta M, Orchard T, Fuller J, Parving HH, Bilous R, Chaturvedi N (2008). Effect of candesartan on progression and regression of retinopathy in type 2 diabetes (DIRECT-Protect 2). Lancet.

[23] Ogawa H, Nakayama M, Morimoto T, Uemura S, Kanauchi M, Doi N, Jinnouchi H, Sugiyama S, Saito Y, Japanese Primary Prevention of Atherosclerosis With Aspirin for Diabetes (JPAD) (2008). Trial Investigators. JAMA.

[24] Duckworth W, Abraira C, Moritz T, Reda D, Emanuele N, Reaven PD, Zieve FJ, Marks J, Davis SN, Hayward R, Warren SR, Goldman S, McCarren M, Vitek ME, Henderson WG, Huang GD (2009). Glucose control and vascular complications in veterans with type 2 diabetes. N Engl J Med.

[25] Young LH, Wackers FJ, Chyun DA, Davey JA, Barrett EJ, Taillefer R, Heller GV, Iskandrian AE, Wittlin SD, Filipchuk N, Ratner RE, Inzucchi SE (2009). Cardiac outcomes after screening for asymptomatic coronary artery disease in patients with type 2 diabetes the DIAD study. JAMA.

[26] Home PD, Pocock SJ, Beck-Nielsen H, Curtis PS, Gomis R, Hanefeld M, Jones NP, Komajda M, McMurray JJ (2009). Rosiglitazone evaluated for cardiovascular outcomes in oral agent combination therapy for type 2 diabetes (RECORD): a multicentre, randomised, open-label trial. Lancet.

[27] Frye RL, August P, Brooks MM, Hardison RM, Kelsey SF, MacGregor JM, Orchard TJ, Chaitman BR, Genuth SM, Goldberg SH, Hlatky MA, Jones TL, Molitch ME, Nesto RW, Sako EY, Sobel BE (2009). A randomized trial of therapies for type 2 diabetes and coronary artery disease. N Engl J Med.

[28] Pfeffer MA, Burdmann EA, Chen C-Y, Cooper ME, de Zeeuw D, Eckardt KU, Feyzi JM, Ivanovich P, Kewalramani R, Levey AS, Lewis EF, McGill JB, McMurray JJ, Parfrey P, Parving HH, Remuzzi G, Singh AK, Solomon SD, Toto R (2009). A trial of darbepoetin alfa in type 2 diabetes and chronic kidney disease. N Engl J Med.

[29] Haller H, Ito S, Izzo JL, Januszewicz A, Katayama S, Menne J, Mimran A, Rabelink TJ, Ritz E, Ruilope LM, Rump LC, Viberti G, ROADMAP Trial Investigators (2011). Olmesartan for the delay or prevention of microalbuminuria in type 2 diabetes. N Engl J Med.

[30] Bland JM, Kerry SM (1998). Statistics notes. Weighted comparison of means. BMJ.

[31] Solomon SD (2010). Cardiovascular clinical trials in patients with diabetes mellitus: lessons from the Bypass Angioplasty Revascularization Investigation 2 Diabetes (BARI 2D) Study. Circulation.

[32] Adler AI, Stevens RJ, Manley SE, Bilous RW, Cull CA, Holman RR (2003). Development and progression of nephropathy in type 2 diabetes: the United Kingdom Prospective Diabetes Study (UKPDS 64). Kidney Int.

[33] Gaede P, Lund-Andersen H, Parving HH, Pedersen O (2008). Effect of a multifactorial intervention on mortality in type 2 diabetes. N Engl J Med.

[34] Yusuf S, Sleight P, Pogue J, Bosch J, Davies R, Dagenais G (2000). Effects of an angiotensin-converting–enzyme inhibitor, ramipril, on cardiovascular events in high-risk patients. N Engl J Med.

[35] Vlagopoulos PT, Tighiouart H, Weiner DE, Griffith J, Pettitt D, Salem DN, Levey AS, Sarnak MJ (2005). Anemia as a risk factor for cardiovascular disease and all-cause mortality in diabetes: the impact of chronic kidney disease. J Am Soc Nephrol.

[36] Desai AS, Toto R, Jarolim P, Uno H, Eckardt KU, Kewalramani R, Levey AS, Lewis EF, McMurray JJ, Parving HH, Solomon SD, Pfeffer MA (2011). Association between cardiac biomarkers and the development of ESRD in patients with type 2 diabetes mellitus, anemia, and CKD. Am J Kidney Dis.

[37] McMurray JJ, Uno H, Jarolim P, Desai AS, de Zeeuw D, Eckardt KU, Ivanovich P, Levey AS, Lewis EF, McGill JB, Parfrey P, Parving HH, Toto RM, Solomon SD, Pfeffer MA (2011). Predictors of fatal and nonfatal cardiovascular events in patients with type 2 diabetes mellitus, chronic kidney disease, and anemia: an analysis of the Trial to Reduce cardiovascular Events with Aranesp (darbepoetin-alfa) Therapy (TREAT). Am Heart J.

[38] Seshasai SR, Kaptoge S, Thompson A, Di Angelantonio E, Gao P, Sarwar N, Whincup PH, Mukamal KJ, Gillum RF, Holme I, Njølstad I, Fletcher A, Nilsson P, Lewington S, Collins R, Gudnason V, Thompson SG, Sattar N, Selvin E, Hu FB, Danesh J, Emerging Risk Factors Collaboration (2011). Diabetes mellitus, fasting glucose, and risk of cause-specific death. N Engl J Med.

[39] Preiss D, Sattar N, McMurray JJ (2010). Event rates in trials of patients with type 2 diabetes. JAMA.

[40] Preiss D, Sattar N, McMurray JJ (2011). A systematic review of event rates in clinical trials in diabetes mellitus: the importance of quantifying baseline cardiovascular disease history and proteinuria and implications for clinical trial design. Am Heart J.

[41] Sarwar N, Gao P, Seshasai SR, Gobin R, Kaptoge S, Di Angelantonio E, Ingelsson E, Lawlor DA, Selvin E, Stampfer M, Stehouwer CD, Lewington S, Pennells L, Thompson A, Sattar N, White IR, Ray KK, Danesh J (2010). The Emerging Risk Factors Collaboration. Diabetes mellitus, fasting blood glucose concentration, and risk of vascular disease: a collaborative meta-analysis of 102 prospective studies. Lancet.

[42] Kovesdy CP, Anderson JE (2007). Reverse epidemiology in patients with chronic kidney disease who are not yet on dialysis. Semin Dial.

[43] Trevisan R, Vedovato M, Mazzon C, Coracina A, Iori E, Tiengo A, Del Prato S (2002). Concomitance of diabetic retinopathy and proteinuria accelerates the rate of decline of kidney function in type 2 diabetic patients. Diabetes Care.

